# Recent advancements in the structural biology of human telomerase and their implications for improved design of cancer therapeutics

**DOI:** 10.1093/narcan/zcad010

**Published:** 2023-03-03

**Authors:** Griffin A Welfer, Bret D Freudenthal

**Affiliations:** Department of Biochemistry and Molecular Biology, University of Kansas Medical Center, Kansas City, KS 66160, USA; University of Kansas Cancer Center, Kansas City, KS 66160, USA; Department of Biochemistry and Molecular Biology, University of Kansas Medical Center, Kansas City, KS 66160, USA; Department of Cancer Biology, University of Kansas Medical Center, Kansas City, KS 66160, USA; University of Kansas Cancer Center, Kansas City, KS 66160, USA

## Abstract

Telomerase is a specialized reverse transcriptase that synthesizes telomeric repeats at the ends of linear chromosomes. Telomerase is transiently expressed in germ and stem cells, but nearly all somatic cells silence it after differentiating. However, the vast majority of cancer cells reactivate and constitutively express telomerase to maintain replicative immortality. Because of this, telomerase has remained a promising broad-spectrum chemotherapeutic target for over 30 years. However, various challenges associated with obtaining high-resolution structural data for telomerase have limited the development of rationally designed structure-based therapeutics. Various techniques and model systems have been utilized to advance our understanding of the structural biology of telomerase. In particular, multiple high-resolution cryogenic electron microscopy (cryo-EM) structures published within the past few years have revealed new components of the telomerase complex with near atomic resolution structural models. Additionally, these structures have provided details for how telomerase is recruited to telomeres and its mechanism of telomere synthesis. With these new pieces of evidence, and the promising outlook for future refinements to our current models, the possibility of telomerase specific chemotherapeutics is becoming more tangible than ever. This review summarizes these recent advancements and outlines outstanding questions in the field.

## INTRODUCTION

**Figure 1.**
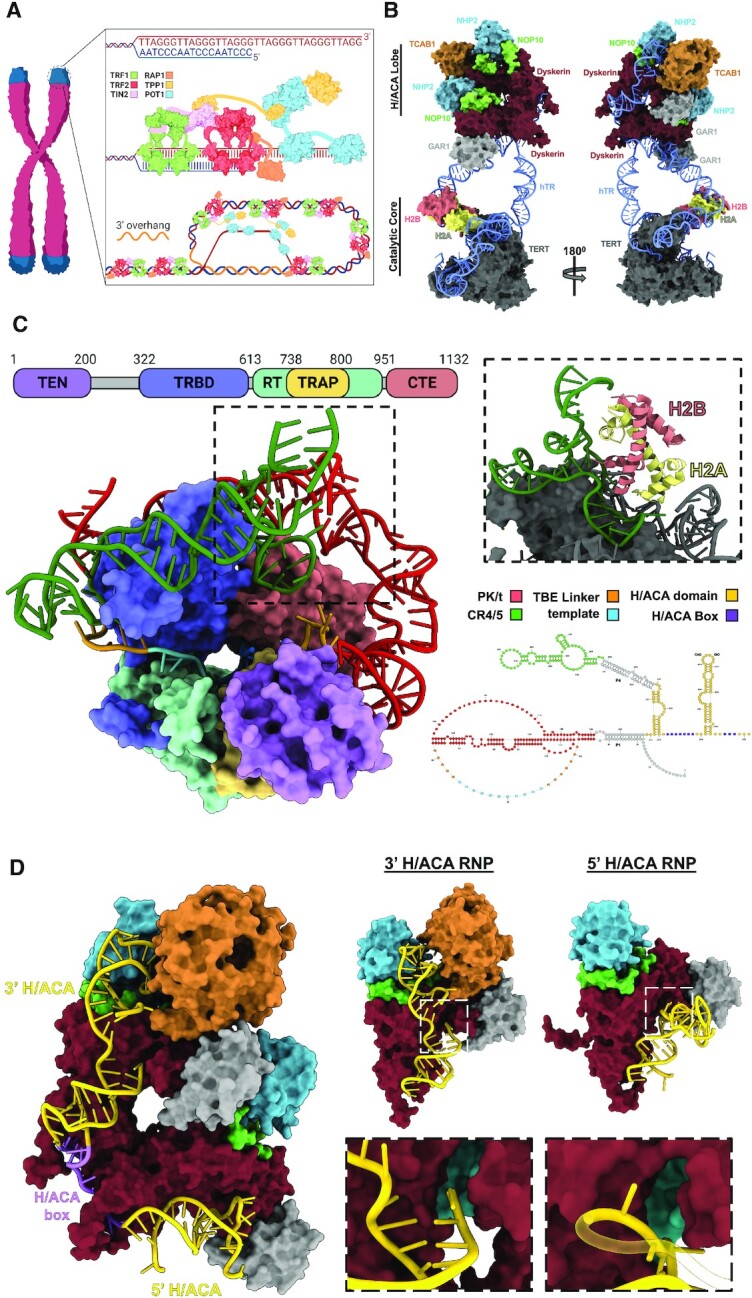
Overall structure of human telomerase. (**A**) Architecture of human telomeres. Human telomeres are composed of tandem TTAGGG repeats that terminate in a 3′ single-stranded DNA overhang. Telomeric DNA is bound by a six-protein complex called shelterin. The shelterin components TRF1 and TRF2 bind to double-stranded telomeric repeats, and POT1 binds to the 3′ overhang. TPP1 does not bind DNA but is localized to the telomere because it forms a heterodimer with POT1. The overall shelterin complex is tethered by TIN2, and RAP1 interacts with TRF2 near the end of the telomeric duplex DNA. The shelterin complex sculpts the telomere into a t-loop (bottom figure in inset) that masks the chromosome 3′ end by forming a D-loop with upstream telomeric DNA. (**B**) Structure of the human telomerase holoenzyme (PDB: 7BG9 (catalytic core), and PDB:7BGB (H/ACA lobe)). (**C**) Domain architecture of hTERT. (Inset: interaction of histone H2A-H2B dimer with CR4/5 of hTR). Secondary structure and domain organization of hTR. (**D**) Structure of H/ACA lobe. Organization of the 3′ H/ACA RNP (inset: autoinhibitory hairpin of hTR in the dyskerin active site (turquoise)). Organization of the 5′ H/ACA RNP (inset: autoinhibitory hairpin of hTR in the dyskerin active site (turquoise)).

Telomeres are nucleoprotein structures that cap the ends of linear chromosomes (1). Human telomeres are typically composed of between 2 and 10 kb of tandem TTAGGG repeats that end in a 3′ single-stranded DNA overhang (Figure 1A) (2,3). Telomeric DNA is coated by a six-protein complex called shelterin that sculpts telomeres into a lariat structure called a t-loop. The t-loop is formed by the 3′ single-stranded DNA overhang invading into an upstream double-stranded DNA segment of the telomere (Figure 1A). The architecture of the t-loop masks the end of the chromosome and prevents it from activating the DNA damage response (4–7). Long telomeres form a protective barrier for the chromosome, but incomplete replication of the lagging strand causes progressive telomere shortening with each cell division (8). In contrast, critically shortened telomeres aren’t effectively bound by shelterin, and trigger a DNA damage response that culminates in a state of permanent cell cycle arrest called replicative senescence (9–11). Therefore, long term maintenance of genomic integrity in highly proliferative tissues such as germline and stem cells requires a mechanism to replenish lost telomeric DNA. Accordingly, cells have evolved a specialized reverse transcriptase called telomerase to maintain their telomeres via *de novo* synthesis of telomeric repeats (12–15).

## STRUCTURE OF HUMAN TELOMERASE

Human telomerase is a ribonucleoprotein (RNP) complex composed of the catalytic protein subunit telomerase reverse transcriptase (TERT), the telomerase RNA component (hTR), and an H/ACA RNP consisting of a single copy of TCAB1 and two copies each of dyskerin, GAR1, NHP2 and NOP10 (14,16). Recent high-resolution structures have also identified a subset of telomerase complexes contain two histone proteins, H2A and H2B, which form a H2A-H2B dimer bound to hTR (17–20). The holoenzyme is arranged in a bilobal architecture bridged by hTR with TERT and H2A–H2B occupying the catalytic core lobe and the H/ACA RNP occupying the other (Figure 1B) (16–19,21).

The catalytic core is responsible for telomere synthesis via TERT. TERT has four domains: the telomerase essential N-terminal domain (TEN), the telomerase RNA binding domain (TRBD), the reverse transcriptase domain (RT), and the C-terminal extension (CTE) (16). Additionally, structural studies of telomerase from the ciliate *Tetrahymena thermophila* discovered a subdomain within the RT domain called TRAP, which forms an interface with the TEN domain to facilitate telomerase processivity by pinning hTR to TERT (22,23). Subsequent bioinformatic analysis of TERT sequences and high-resolution structures confirmed TRAP is conserved in human telomerase (Figure 1C) (23). The pseudoknot/template (PK/t) and conserved regions 4 and 5 (CR4/5) of hTR encircle and stabilize TERT (Figure 1C, Supplemental Movie 1) (17). The PK/t domain flanks both ends of the template motif with flexible single-stranded linkers that define the boundaries of the template sequence and facilitate movement of the template during nucleotide addition (Figure 1C) (24).

The functional role of the H2A–H2B dimer is unclear, but its presence in ∼60% of particles suggests it is significant for assembly of active telomerase (18). These structures indicate the H2A–H2B dimer uses the same positively charged surface it uses to bind nucleosomal DNA to bind and stabilize the three-way junction within the CR4/5 region of hTR (Figure 1C, Supplemental Movie 1). The La-related protein p65 forms similar interactions with the three-way junction of *Tetrahymena thermophila's* telomerase RNA component, which suggests the H2A–H2B interaction may be functionally similar (17,25). Notably, all four histone proteins of the nucleosome core particle (H2A, H2B, H3 and H4) were identified as telomerase components via immunoblotting and mass spectrometry, but only H2A and H2B immunoprecipitated samples showed detectable levels of telomerase activity (18). It's possible that H3 and H4 are too flexible to be resolved in structures of telomerase; however, no known structures of the histone octamer are compatible with the current structures of telomerase containing an H2A-H2B dimer. While there is substantial evidence for the presence of an H2A–H2B dimer in overexpressed telomerase complexes (17–20), they are likely a substoichiometric component of the endogenous complex as immunoblots from endogenous telomerase complexes only faintly detected the four histone proteins (H2A, H2B, H3 and H4) (18). Future investigations into the functional roles of H2A, H2B, H3 and H4 in human telomerase will surely produce interesting findings. Presently, existing evidence supports a role for the H2A-H2B dimer as a folding cochaperone during telomerase maturation, with unclear effects of their presence on overall enzyme activity.

The second component of telomerase is the H/ACA lobe, which contains 5′ and 3′ hairpins separated by an H/ACA box between the interface of the dyskerin dimer (Figure 1D, Supplemental Movie 2). Human telomerase was the first structure of a eukaryotic H/ACA RNP and revealed the general architecture used by other H/ACA RNPs (16). Canonical H/ACA RNPs are responsible for pseudouridylation of cellular RNAs (26). However, human telomerase has not been shown to have pseudouridine synthase activity. The basis for this is due to two hairpins within hTR blocking the pseudouridylation pocket of both dyskerin subunits within the H/ACA lobe, thus preventing canonical substrate binding (Figure 1D) (17). Therefore, telomerase's H/ACA RNP functions as a key regulatory component for trafficking and maturation, rather than a catalytic component.

## MECHANISM OF TELOMERE SYNTHESIS BY HUMAN TELOMERASE

**Figure 2.**
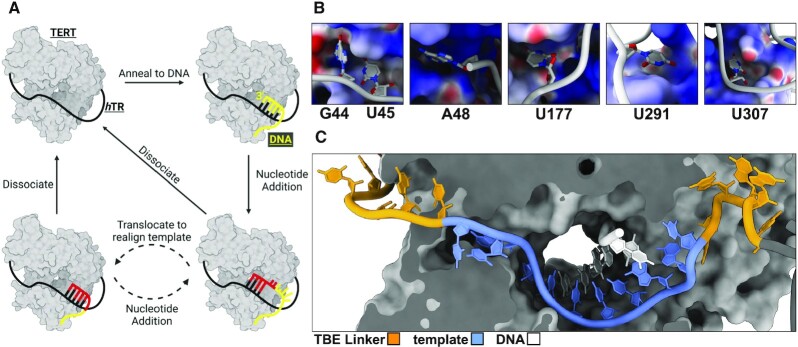
Telomerase mechanism of telomere synthesis. (**A**) Generalized model of TERTs mechanism of telomere synthesis. Telomerase uses hTR to anneal to the 3′ end of the telomere (yellow). Next, TERT catalyzes the consecutive addition of up to six nucleotides to the chromosome end using the template sequence of hTR. After synthesizing a complete telomeric repeat (red), TERT can translocate along the DNA to realign the newly extended telomere with the 5′ end of its template in order to synthesize an additional six nucleotides. The cyclic process of nucleotide addition followed by translocation is called repeat addition processivity (RAP). (**B**) Specific interactions between labeled residues of hTR and surface pockets of TERT (TERT is colored by its surface electrostatic environment. Palette: red: negatively charged, white: neutral, blue: positively charged). (**C**) path of the DNA, template, and template boundary element regions of hTR within TERTs active site.

Telomerase has a unique catalytic cycle among known DNA polymerases and reverse transcriptases because it utilizes an internal RNA motif embedded within hTR as a template to direct telomere synthesis (Figure 2A). First, telomerase uses its RNA template to anneal to the 3′ end of a telomere. TERT then sequentially adds up to six nucleotides to the chromosome before reaching the end of its template. After synthesizing a complete telomeric repeat, telomerase can translocate along the DNA to realign the newly extended telomere back in its active site to undergo another six rounds of nucleotide addition. The cyclic process of nucleotide addition followed by translocation is called repeat addition processivity (RAP) and is a telomerase specific phenomenon (27).

Along with uncovering the structural composition of telomerase, high resolution structures are amenable to computer simulation studies, which can be used to derive dynamic functional insight into telomerase's mechanism of telomere synthesis. For example, the length of the DNA/RNA duplex within the TERT active site is maintained between 3 and 5 nucleotides, implicating significant stabilizing contacts contributed by TERT and hTR to prevent the duplex from melting (17,25). Wan et. al. determined how telomerase maintains this unusually short duplex using molecular dynamics simulations. These simulations revealed that a single residue (Leu980) acts as a wedge that unzips the DNA/RNA duplex as TERT translocates across its template. Consistent with this, mutating this residue to a glycine increased the duplex length and reduced telomerase processivity. These simulations also revealed that without hTR, TERT is significantly more dynamic. Specifically, they identified the PK/t and CR4/5 regions of hTR stabilize TERT and improve the concerted movement of each of its four domains (17). The hTR/TERT interactions include RNA backbone interactions with hydrophilic surface residues as well as specific anchor positions where nucleobases of hTR sit within pockets on TERTs surface (Figure 2B) (17). The interfaces between TERT and hTR may be promising candidates for therapeutic targeting as disrupting these interactions would specifically innhibit telomerase without off target effects on other DNA and RNA polymerases. Future analyses using computational techniques will likely provide additional mechanistic details into the functional roles of residues involved in telomerase's mechanism that may then be used to guide therapeutic drug development.

Every structure of human telomerase solved thus far has been at the same registry with hTR. In each of these structures A48 of hTR is directly 5′ of the templating base used for nucleotide addition. However, structures of *Tetrahymena* telomerase at different registries suggest that the flipped positioning of the base immediately 5′ of the templating position is most likely shared by other residues of hTR when they occupy this position (25). Following nucleotide addition and translocation this base will flip into a templating orientation for catalysis (25). These structures of *Tetrahymena* telomerase at different registries confirmed that TERT does not undergo significant rearrangements as it progresses along the template (25,28). Instead, the linker regions flanking the template motif undergo significant stretching and movement at different stages of telomere elongation to define the template sequence boundaries (Figure 2C, Supplemental Movie 3).

## TELOMERASE RECRUITMENT TO TELOMERES

**Figure 3.**
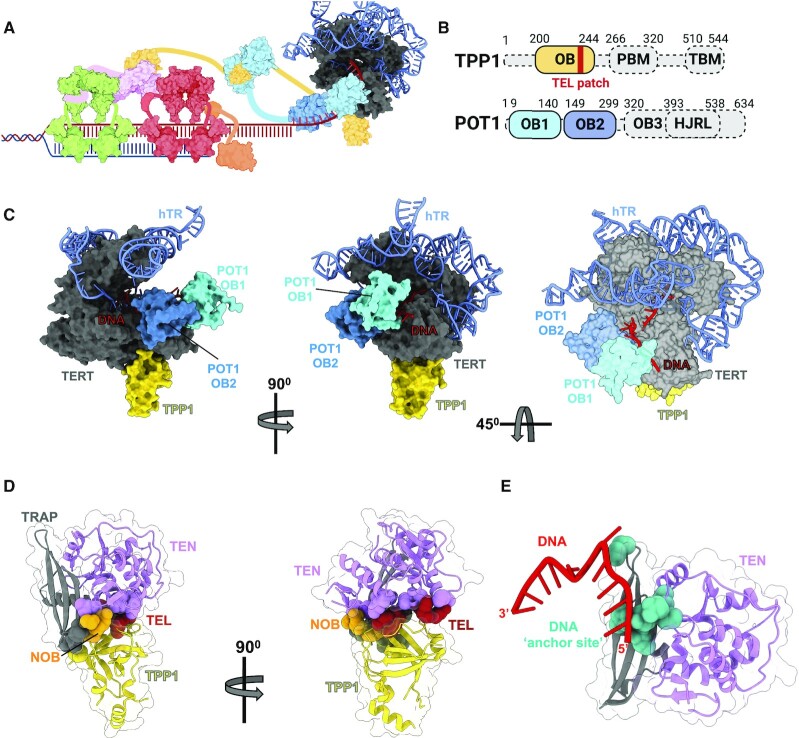
Structural basis of telomerase recruitment and retention at telomeres. (**A**) Schematic of how telomerase interacts with the shelterin proteins TPP1-POT1 at the chromosome end. (**B**) Domain architecture of TPP1 and POT1 (domains resolved in cryo-EM structures of telomerase's catalytic core in complex with TPP1 or TPP1-POT1 are colored as in panel C. Unresolved domains are grayed out. (**C**) Structure of telomerase's catalytic core in complex with TPP1-POT1 (PDB: 7QXB). (**D**) Interactions between the TEL patch (red) and NOB region (orange) of TPP1 with the TEN-TRAP interface of TERT. (**E**) Putative telomerase ‘anchor site’ responsible for maintaining contact with DNA during RAP.

Telomerase expression is tightly controlled and even cancer cells maintain its levels around a 2:1 ratio with the number of telomeres following DNA replication (∼250 copies per cell) (29). Given these low levels and the overall size of the genome, the mechanism of telomerase recruitment to telomeres has been a longstanding question in the field (30). Biochemical and genetic studies have implicated the shelterin protein TPP1 in telomerase recruitment to telomeres (31). TPP1 assembles with the shelterin components POT1 and TIN2, and the TPP1–POT1–TIN2 complex has been extensively shown to both recruit telomerase and stimulate RAP (32). Recent high-resolution structures of telomerase bound to TPP1 and TPP1–POT1 have provided insight into the structural basis of telomerase recruitment (16,20). These structures revealed how the oligonucleotide/oligosaccharide binding domain (OB) of TPP1 and the OB1 and OB2 domains of POT1 interact with TERT to facilitate RAP (Figure 3A). Notably only a subset of the TPP1–POT1–TIN2 domains were resolvable (Figure 3B, C) (19). This is consistent with recent efforts to determine the structure of the shelterin complex, which have demonstrated each of the subunits are highly dynamic and difficult to resolve (33–35). The dynamics of shelterin likely play a key role in protecting telomeric DNA and the stabilization of TPP1–POT1 may be required for effective telomerase recruitment (36). Previous structures of telomerase have consistently shown that the TEN domain adopts the most conformational variability among the TERT domains. However, these structures revealed TPP1 uses its N-terminal OB-fold domain (NOB) to stabilize the TEN domain. NOB sits in a hydrophobic pocket between the TRAP–TEN interface, a glutamate-rich region within TPP1 called the TEL patch resides in a basic surface on the TEN domain, and additional residues within the TEL patch form extensive interactions across the TRAP-TEN domains (Figure 3D). These structures also contained density for a 12-nucleotide chain of DNA emerging from the 5′ end of TERT’s active site, providing evidence for the path of emerging DNA following telomere synthesis (Figure 3C). While there is no electron density for DNA beyond 12 bp in the structure of telomerase bound to TPP1–POT1, modeling DNA from a crystal structure of POT1 was used to create a proposed path for nearly three complete telomeric repeats (Figure 3C). Tracing the DNA path from its 5′ end into the TERT active site shows the DNA is encircled by a positively charged tunnel formed between POT1’s OB1 domain and the TEN domain of TERT (Figure 3C). The DNA is then positioned along the TEN domain via POT1’s OB2 domain and traverses across the TRAP and CTE domains before entering the TERT active site. The extensive interactions between TERT–TPP1–POT1 and the DNA provide a physical explanation for telomerase's ability to undergo RAP, where the DNA product must disengage the hTR RNA template without completely dissociating from the telomerase holoenzyme. Highly conserved motifs within the TEN domain (residues 174–177) and TRAP domain (residues 752–759 and 794) facilitate the 5′ DNA path and are likely the elusive DNA ‘anchor site’ proposed to maintain contact with the telomeric DNA during translocation of telomerase (Figure 3E).

## PERSPECTIVE FOR CANCER THERAPIES

Structure-based drug design (SBDD) is a computational approach used to predict interactions between a drug candidate and its target protein. SBDD uses the three-dimensional structure of the target protein to design and optimize drug candidates that target specific sites on a protein to modulate its activity. This approach aims to minimize non-specific interactions between the drug and other macromolecules to maximize the effectiveness and potency of a drug candidate. Telomerase's heterogenous composition and configurational complexity, which had plagued early attempts to solve its structure, are finally beginning to be overcome thanks to technological improvements in cryo-EM structural determination. Continued improvements in data processing software will allow many of the heterogenous complexes to be resolved which may uncover new functional interactions and binding partners that have yet to be determined. However, the exact positions of amino acid sidechains and mobile nucleic acid elements may remain ambiguous without very high-resolution structures. Therefore, high resolution crystallographic or cryo-EM structures with well resolved sidechains are still required to characterize the specific molecular details of telomerase's activity, particularly within its active site during nucleotide addition. However, with near atomic resolution models, telomerase is finally becoming amenable to SBDD techniques.

Identifying possible telomerase drug targets, can be assisted by analyzing the large number of disease-associated mutations found across the components of the telomerase holoenzyme (http://telomerase.asu.edu/). Mapping these mutations onto its structure reveals important interactions that may be targetable for telomerase inhibition (16,18). Many of these mutations are found at residues that interact with hTR, within interdomain contacts, and at essential active site residues (18,20). Additionally, utilizing computer simulations to characterize the mechanism of telomere synthesis could provide potent therapeutic targets. For example, the small 3–4 bp duplex must be stabilized through interactions with TERT and understanding the physical basis of this stabilization may provide a basis for disrupting telomerase activity. Consistent with this, therapeutic nucleotide analogs and different forms of DNA damage that can perturb DNA structure have been shown to disrupt telomerase processivity (37). Furthermore, since various telomerase inhibitors have been developed and investigated biochemically for decades, solving structures of these molecules bound to telomerase can assist in rational design of next generation drug candidates. An example of this includes the highly specific telomerase inhibitor BIBR1532, which interferes with a critical hTR/hTERT interface (20,38). While BIBR1532 was identified from biochemical screens, subsequent structures of it bound to TERT have enabled the design of next generation molecules with increased potency (39,40). A recent example of leveraging structural information to define the mechanism of action for a therapeutic molecule was recently demonstrated for remdesivir stalling the RNA dependent RNA polymerase (RdRp) of SARS-CoV-2. Cryo-EM structures of the RdRp stalled following remdesivir insertion revealed multiple remdesivir insertions lead to a delayed translocation inhibition effect (41). Analogous structures of telomerase bound to translocation inhibitors may shed light on their specific mechanism of action and be used to guide design of additional therapeutics. The fundamental mechanism behind telomerase's RAP activity still needs to be clearly defined. Recent structures of tetrahymena telomerase at different stages of nucleotide addition have revealed many details for this process in ciliates, and similar structures of telomerase engaged during various points of nucleotide insertion will be required to identify conserved mechanistic details as well as any possible vertebrate specific features (25). Designing small molecules that interfere with the TPP1–POT1 and TERT interfaces that would prevent telomerase recruitment may also be a viable strategy, as mutations in TPP1 or POT1 have been shown to change telomerase's recruitment and retention (42).

The design of novel therapeutics will likely be expedited through improved computational techniques for drug development. This line of reasoning is based on the revolutionary power of modern machine learning (ML) and artificial intelligence (AI). ML and AI’s ability to solve some of the most difficult problems in molecular biology has been demonstrated with the success of protein structure prediction programs such as AlphaFold (43). While ML and AI have been fundamental components of recent structure-based drug design algorithms, continued improvements in computing capabilities will likely lead to similar advancements for efficient drug design (44–48). While a systematic overview of the process of structure based drug design is beyond the scope of the current work, we direct the reader to these excellent reviews on general strategies for structure based drug design as well as how it has been applied to identify therapeutic compounds targeting SARS-CoV-2 (44,47,49,50). Collectively, the recent advancements in our understanding of telomerase's structure have come at a promising time for structure-based drug design. Meaning we may finally hit a ‘bullseye’ with this historically elusive target.

## DATA AVAILABILITY

No new data were generated or analysed in support of this research.

## Supplementary Material

zcad010_Supplemental_Files
