# Supplementary material for: Recent advancements in the structural biology of human telomerase and their implications for improved design of cancer therapeutics
Source: NAR Cancer. 2023 Mar 3;5(1):zcad010. doi: 10.1093/narcan/zcad010 (PMC9984990; doi:10.1093/narcan/zcad010)
Supplement: zcad010_Supplemental_Files [file zcad010_supplemental_files.zip › Supplemental Movie legends.docx]

**Supplemental Movie 1)** Structure of human telomerase catalytic core (PDB: 7BG9).

**Supplemental Movie 2)** Structure of human telomerase H/ACA lobe (PDB: 7BGB).

**Supplemental Movie 3)** Morph of tetrahymena telomerase from position +3 (PDB: 7LMA) to +5 (PDB: 7LMB).
